# Monitoring the Early Antiproliferative Effect of the Analgesic–Antitumor Peptide, BmK AGAP on Breast Cancer Using Intravoxel Incoherent Motion With a Reduced Distribution of Four *b*-Values

**DOI:** 10.3389/fphys.2019.00708

**Published:** 2019-06-21

**Authors:** Natacha Raissa Doudou, Sylvanus Kampo, Yajie Liu, Bulbul Ahmmed, Dewei Zeng, Minting Zheng, Aminou Mohamadou, Qing-Ping Wen, Shaowu Wang

**Affiliations:** ^1^Department of Radiology, Dalian Medical University, Dalian, China; ^2^Department of Radiology, The Second Affiliated Hospital of Dalian Medical University, Dalian, China; ^3^Department of Anesthesiology, Dalian Medical University, Dalian, China; ^4^Department of Anesthesiology, The First Affiliated Hospital of Dalian Medical University, Dalian, China; ^5^Department of Biochemistry and Molecular Biology, Liaoning Provincial Core Lab of Glycobiology and Glycoengineering, Dalian Medical University, Dalian, China; ^6^Department of Radiology, The First Affiliated Hospital of Dalian Medical University, Dalian, China

**Keywords:** intravoxel incoherent motion, analgesic–antitumor peptide, *Buthus martensii* Karsch, magnetic resonance imaging, breast neoplasms

## Abstract

**Background:** The present study aimed to investigate the possibility of using intravoxel incoherent motion (IVIM) diffusion magnetic resonance imaging (MRI) to quantitatively assess the early therapeutic effect of the analgesic–antitumor peptide BmK AGAP on breast cancer and also evaluate the medical value of a reduced distribution of four *b*-values.

**Methods:** IVIM diffusion MRI using 10 *b*-values and 4 *b*-values (0–1,000 s/mm^2^) was performed at five different time points on *BALB/c* mice bearing xenograft breast tumors treated with BmK AGAP. Variability in *Dslow*, *Dfast*, *PF*, and *ADC* derived from the set of 10 *b*-values and 4 *b*-values was assessed to evaluate the antitumor effect of BmK AGAP on breast tumor.

**Results:** The data showed that *PF* values significantly decreased in rBmK AGAP-treated mice on day 12 (*P* = 0.044). *PF* displayed the greatest AUC but with a poor medical value (AUC = 0.65). The data showed no significant difference between IVIM measurements acquired from the two sets of *b*-values at different time points except in the *PF* on the day 3. The within-subject coefficients of variation were relatively higher in *Dfast* and *PF*. However, except for a case noticed on day 0 in *PF* measurements, the results indicated no statistically significant difference at various time points in the rBmK AGAP-treated or the untreated group (*P* < 0.05).

**Conclusion:** IVIM showed poor medical value in the early evaluation of the antiproliferative effect of rBmK AGAP in breast cancer, suggesting sensitivity in *PF*. A reduced distribution of four b-values may provide remarkable measurements but with a potential loss of accuracy in the perfusion-related parameter *PF*.

## Introduction

Breast cancer remains the second leading cause of cancer-related deaths among women (Siegel et al., 2017). Breast cancer includes various histological groups classified in four main molecular subtypes: human epithelial growth receptor type 2 (HER-2), luminal A, luminal B, and basal-like breast cancer subtype (Tong et al., 2018). According to the histological subtype, breast cancer at a particular stage is likely to metastasize to some parts of the body, mostly to the lungs, the brain, and other organs such as the bones, the liver, or soft tissues (Kennecke et al., 2010). Each biological subtype presents different patterns translating its aggressiveness (Kennecke et al., 2010). Although recent approaches improved the diagnosis of breast cancer by giving interesting complementary information, like the evaluation of immunohistochemical biomarkers HER-2, progesterone receptors (PRs), estrogen receptors (ERs), and proliferation status (Ki-67) generally used to establish the prognosis and the predictive factors for the disease (Karlsson et al., 2014), the complex nature of these tumors mostly presents a significant challenge in therapeutic approaches.

Triple-negative breast cancer lacks well-defined molecular targets and has few effective options for treatment. Chemotherapy remains the therapeutic mainstay which sometimes gives an exciting and complete response to therapy. Unfortunately, this alternative does not prevent patients from a high possibility of recurrence and metastases (Liedtke et al., 2008). The poor clinical prognosis associated with breast cancer, therefore, reinforced the urgent need for effective alternative therapeutic options. Recently, several types of research were carried out around a targeted treatment with various pathways (Al-Mahmood et al., 2018; Moore-Smith et al., 2018; Rodríguez Bautista et al., 2018; Tong et al., 2018). Advances in genomic studies and scorpion venom provided encouraging results for their potential use in clinical cancer management (Sarfo-Poku et al., 2016). The Chinese scorpion, *Buthus martensii* Karsch (BmK), venom is a natural compound that contains mixtures of peptides that have analgesic and antitumor properties. Many studies reported some physiological effects of the Chinese scorpion BmK venom known for its antiepileptic, analgesic, and, recently, antitumor properties. The use of this Chinese scorpion BmK venom revealed apoptogenic, immunosuppressive, and antiproliferative properties and demonstrated the potential of the cocktail for cancer-related diseases (Zhao et al., 2011; Li et al., 2014; Al-Asmari et al., 2016, 2018; Guo et al., 2016; Liu et al., 2017). The antitumor effect of the BmK venom was later accredited to an analgesic peptide called BmK AGAP and others (Zhao et al., 2011; Guo et al., 2016).

Diffusion-weighted imaging (DWI), magnetic resonance imaging (MRI), and immunohistochemistry analyses have displayed potentials as clinical biomarkers essential for the assessment of responses to treatment and the monitoring of tumor progression in patients (Patterson et al., 2008; Zhuang et al., 2018). Intravoxel incoherent motion (IVIM), the bi-exponential model of diffusion proposed by Le Bihan can quantify the incoherent signals in each voxel of the tumors and display three parameters: the molecular “true” diffusion coefficient (*Dslow*), the microvascular volume fraction (*PF*), and the perfusion-related incoherent microcirculation (*Dfast*) (Le Bihan et al., 1986).

Previous studies have demonstrated that diffusion parameters could provide accurate information on tumor cellularity (Sugahara et al., 1999; Guo et al., 2002; Fan et al., 2005; Humphries et al., 2007; Yamashita et al., 2009; Iima et al., 2014; Robba et al., 2017). Other studies have associated low pretreatment diffusion values with lesion aggressiveness and malignancy, whereas high values predict inadequate response to therapy with eventual necrosis and cystic components in a tumor (Koh et al., 2007; Cui et al., 2008; Turkbey et al., 2011). Despite the existing debate on standardization of the appropriate number of *b*-values convenient for IVIM analysis, the abilities of the molecular “true” diffusion coefficient (*Dslow*), the microvascular volume fraction (*PF*), and the perfusion-related incoherent microcirculation (*Dfast*) to measure physiological and pathological changes in the living tissues have been widely applied to assess and predict the responses to neoadjuvant chemotherapies in breast cancer (Che et al., 2016; Cho et al., 2017; Kim et al., 2018). The present study aimed to assess the potential medical value of IVIM-MRI-derived parameters in monitoring early changes in breast tumor treated with rBmK AGAP and if the measurements acquired from the set of fewer *b*-values (4 *b*-values) are similar in accuracy to a greater selection of 10 *b*-values.

## Materials and Methods

### Cell Culture

MDA-MB-231 cells were purchased from the American Type Culture Collection (Beijing Zhongyuan Limited, China). Using short tandem repeat (STR) analysis, the MDA-MB-231 cells were authenticated by Beijing Microread Genetics (Beijing, China) before purchase. The MDA-MB-231 cells were consistently maintained in high-glucose Dulbecco’s modified Eagle’s medium (DMEM) (Gibco, United States), supplemented with 10% fetal bovine serum (Gibco, United States), penicillin 100 units/ml, and streptomycin 100 μg/ml (TransGen Biotech, China). The cells were cultured in an incubator at 37°C humidified air with 5% CO_2_ atmospheric condition. The MDA-MB-231 cells were routinely subcultured every 3–5 days.

### Preparation of Recombinant BmK AGAP

Shenyang Pharmaceutical University School of Life Sciences and Bio-pharmaceutics (Shenyang, China) provided the recombinant BmK AGAP (rBmK AGAP) for this study. The rBmK AGAP was obtained as formerly described in a previous study (Ma et al., 2010). The rBmK AGAP solution was diluted with 0.9% saline and filtered with a 0.22-μm sterile membrane before use. The activity of the rBmK AGAP was the same as in the previous study.

### Cell Viability and Toxicity Assay of rBmK AGAP

The inhibitory concentration value (IC_50_) of rBmK AGAP was evaluated using 3-(4-5-dimethylhiazol-2-yl)-2,5-diphenyltetrazolium bromide (MTT) assay. MDA-MB-231 cells were seeded in 96-well plastic plates at a density of 1 × 10^4^ cells per well and incubated at 37°C overnight. Different concentrations of rBmK AGAP (0, 5, 10, 20, 40, 60, and 80 μM) were then used to treat the cells and incubated in a humidified atmosphere of 5% CO_2_ at 37°C for 48 h. Saline (0.9%) was added to the untreated cells as the control groups. Then, 20 μl of MTT stock solution (5 mg/ml) was added to each well, and cells were incubated for an additional 4 h at 37°C. The MTT solution was then discarded, and 100 μl of dimethyl sulfoxide (DMSO) solution was added into each well and incubated for 30 min in the dark to dissolve the insoluble formazan crystals. Optical density (OD) was measured at a test wavelength of 590 nm and a reference wavelength of 650 nm using an enzyme-linked immunosorbent assay (ELISA) multiwell plate reader (BioTek Instruments, Winooski, VT, United States). To calculate percentage cell viability, OD values were used in the formula:

$$\%\,\mathrm{of}\,\mathrm{cell}\,\mathrm{viability}=\frac{\mathrm{OD}\,\mathrm{value}\,\mathrm{of}\,\mathrm{experimental}\,\mathrm{sample}\,\left(\mathrm{treated}\,\mathrm{cells}\right)}{\mathrm{OD}\,\mathrm{value}\,\mathrm{of}\,\mathrm{experimental}\,\mathrm{control}\,\left(\mathrm{untreated}\,\mathrm{cells}\right)}\times 100.$$

### Mouse Xenograft Tumor Model

The specific pathogen-free (SPF) animal facility of Dalian Medical University provided the experimental animals for this study. The animal ethics committee of the Dalian Medical University, China, approved all the experimental animal procedures. Forty-five female nude *BALB/c* mice between the ages of 6 and 8 weeks and weighing between 18 and 20 g were maintained under sterile conditions during the entire experimental period. The mice were housed in standard transparent plastic cages under 12/12-h light–dark cycle regime and were provided free access to food and water. MDA-MB-231 cells (5 × 10^6^) suspended in 0.2 ml of phosphate-buffered saline (PBS) were injected subcutaneously in the lower right thigh, and after 7 days, the mice were randomly assigned to one of three groups (*n* = 15/group). An equal concentration of rBmK AGAP 0.5 mg/kg or 1 mg/kg of body weight diluted in equal volume (100 μl) of 0.9% saline was injected intraperitoneally for 12 days at 48-h interval. Mice from the untreated group were injected with 0.9% saline. The tumor dimensions were measured every 2 days using a digital caliper (#03000002, GuangLu, China). The tumor volume was calculated according to the formula (Faustino-Rocha et al., 2013):

Volume=1/2Length×Width.2

On days 0, 3, 6, 9, and 12, randomly selected mice bearing the xenograft breast tumor from each group were scanned using non-enhanced MRI sequences including IVIM diffusion. At the end of each scanning time point, the mice were euthanized, breast tumor masses were weighed, and a section of the tumor masses was embedded in paraffin or snap frozen in liquid nitrogen for further experiments.

### Image Acquisition

Magnetic resonance images of each mouse bearing xenograft breast tumor were acquired with 3.0T MRI system (General Electric Discovery MR 750w 3.0T, GE Medical Systems, Waukesha, WI, United States) using an eight-channel high-density dedicated wrist coil array (Invivo Corporation, GE Medical Systems, Gainesville, FL, United States). Each mouse was anesthetized with 5% chloral hydrate (0.1 ml/10 g of body weight) and wrapped in a layer of cotton (∼2 cm thick) to maintain stable body temperature, and then placed in a prone position into an eight-channel high-density dedicated wrist coil (feet first).

Anatomic MRI of each tumor was obtained with T2-weighted image (T2WI) sequence in coronal and axial planes, and an axial T1-fast spin echo (T1-FSE). Axial and coronal T2WIs were acquired using a fat-saturated fast recovery fast spin echo (FRFSE) sequence [10 slices; repetition time (TR) 2,000 ms, echo time (TE) 50 ms, matrix size 192 × 160, field of view (FOV) 8 × 8 cm^2^, slice thickness 1.8 mm, and NEX 6, TA 2 min 04 s]. T1-FSE images were obtained for 10 slices as well as identical to T2WI with the following parameters (TR 450 ms, TE 16.5 ms, matrix size 320 × 256, FOV 8 cm × 8 cm, slice thickness 1.8 mm, and NEX 2, TA 1 min 03 s).

IVIM diffusion MRI was acquired using a free-breathing spin echo echo-planar imaging (SE-EPI) sequence (TR 3,000 ms, TE 98.7 ms, slice thickness 3.6 mm, matrix size 128 × 128, and FOV 8 cm × 8 cm) using diffusion gradients applied in three orthogonal directions and two different combinations of *b*-values: a combination of 10 *b*-values (0, 50, 100, 150, 200, 300, 400, 600, 800, and 1,000 s/mm^2^) and a set of 4 *b*-values [0, 70, 300, and 800 s/mm^2^ (Cho et al., 2015)]. A selective chemical shift saturation technique was used for fat suppression. The total image acquisition time of the MRI sequences was approximately 10 min per each mouse.

### Image Analysis

All the images were transferred into our advantage GE Medical System workstation (AW4.6; GE Medical System). We used the option MADC available on the in-house GE postprocessing Functool software (Functool 9.4.05, GE Medical System) to generate the IVIM parameters [*Dslow*, *Dfast*, *PF*, and apparent diffusion coefficient (*ADC*)]. We assumed the bi-exponential fitting method to generate the IVIM-related parameters with a b threshold at 200 s/mm^2^ using the signal decay formula below:

Sb/S_0_= (1− *PF*). exp (− b*Dslow*) + *PF*.exp (− b*Dfast*).

where Sb stands for the signal intensity present in the pixel when we use a diffusion gradient b and S_0_ is the signal intensity in the pixel without any diffusion gradient (0), *Dslow* represents the “true” diffusion parameter reflecting the pure molecular diffusion, while *Dfast* is the pseudodiffusion coefficient associated with the perfusion effect, and *PF* is the fractional perfusion related to the microcirculation (Le Bihan et al., 1986).

A region of interest (ROI) including the whole tumor was manually drawn using comparative T2WIs and *ADC* maps to locate the tumors. The parameters were expressed as mean values from all pixels within the ROI from the slices.

### Immunohistochemical Staining

Immunohistochemical analysis was performed on paraffin-embedded sections of visible tumors removed from mice. Serial sections (4 μm each) were prepared and stained with hematoxylin–eosin (H&E) for routine histology and Ki-67, vascular endothelial growth factor (VEGF), and nuclear factor-kappa B (NF-κB)/p65 for the analysis of cell proliferation in rBmK AGAP-treated and untreated groups. The 4-μm slices of the tumors were deparaffinized in xylene and rehydrated in graded alcohol. After microwaving for 20 min in citrate buffer to expose antigens, the slides were washed with PBS and incubated in 3% H_2_O_2_ for 10 min at room temperature to block endogenous peroxidase activity. Non-specific binding was blocked with goat serum at room temperature for 30 min before overnight incubation at 4°C with primary antibodies against Ki-67 (#AR53222, Arigo, China), VEGF (Proteintech, China), and NF-κB/p65 (Proteintech, China). After extensive washing with PBS, slides were incubated with biotinylated secondary antibody for 45 min at room temperature. The slides were then incubated with a streptavidin–peroxidase complex. The signal was visualized with DAB (3,3′-diaminobenzidine), and the slides were briefly counterstained with hematoxylin. Yellowish brown stain indicated positive for the antigen of interest. Images of stained tissue slides were captured using a microscope (Olympus BX51, Japan).

### Total RNA Extraction, cDNA Synthesis, and Quantitative Polymerase Chain Reaction

mRNA level was measured with quantitative polymerase chain reaction (qPCR). Nucleospin RNA II isolation kit (Macherey-Nagel, Duren, Germany) was used to extract total RNAs from frozen tissues according to the manufacturer’s protocol. A PrimeScript^TM^ RT reagent kit (TransGen Biotech, China) was used to perform RNA reverse transcription into cDNA, and then qPCR was performed using the TransStart TipTop Green qPCR SuperMix (TransGen Biotech, China) according to the manufacturer’s instructions. The primer sequence for human VEGF were 5′-CTACCTCCACCATGCCAAGT-3′ (F) and 5′-GCAGTAGCTGCGCTGATAGA-3′ (R). The thermal conditions for the qPCR assay in the Applied Biosystems StepOne^TM^ Real-Time PCR thermal cycler (SN-271004043, Thermo Fisher Scientific, United States) were as follows—cycle 1: 95°C for 10 min, cycle 2 (×40): 95°C for 10 s and 58°C for 45 s. Data were normalized to glyceraldehyde 3-phosphate dehydrogenase (GAPDH), and relative quantities were calculated using the 2^–ΔΔCt^ method. Triplicate independent experiments were performed.

### Protein Extraction and Western Blot Analysis

Frozen tissues were lysed in CEB lysis buffer (Invitrogen, Life Technologies, Grand Island, NY, United States). Protein was quantitated by using Pierce’s BCA protein assay reagent kit (from Pieces Biotechnology, Rockford IL, United States) as per the manufacturer’s protocol. After protein extraction from tissues (9 g), equal amounts of proteins (20 μg/well) were separated by 12% sodium dodecyl sulfate–polyacrylamide gel electrophoresis (SDS-PAGE) and transferred to nitrocellulose (NC) membrane guided by a prestained protein molecular weight ladder in a wet transfer system (Beijing Liuyi Biotechnology, China). The membranes were then blocked with 5% fat-free milk in TBST at room temperature for 1 h and probed with primary antibody against Ki-67 (#AR53222, Arigo, China), VEGF (Proteintech, China), NF-κB/p65 (Proteintech, China), IkBα (Proteintech, China), p-p65/NF-κB (Proteintech, China), PARP (Proteintech, China), and GAPDH (Proteintech, China) at 4°C overnight. This was followed by incubation with appropriate secondary antibodies (peroxidase-conjugated goat anti-rabbit IgG, proteintech, China) at room temperature for 1 h. Antibody binding was detected with an enhanced chemiluminescence kit (ECL, Amersham, United Kingdom) and detected in a ChemoDocTMXRS + Imager system (Bio-Rad, United States). Relative quantities were analyzed with Image Lab software v4.0.1 (Bio-Rad, United States).

### Statistical Analysis

Statistical analyses were carried out using SPSS 17.0 version (SPSS statistics 17.0, released on August 23, 2008). All numerical values were depicted as means ± standard deviations (SDs), and the variation coefficients were displayed in percentages. Kolmogorov–Smirnov test was used to evaluate normality in the distributions. We performed independent sample *t*-tests to compare the tumor volumes, the *ADC*, and the IVIM-derived parameters of the different therapeutic groups at various time points during the treatment process. Receiver operating characteristics (ROC) curves were generated from the IVIM-related parameters *Dslow*, *Dfast*, *PF*, and *ADC* values to identify the parameter providing the largest area under the curve (AUC) in the assessment of the rBmk AGAP treatment effect. The results acquired from two combinations of *b*-values were compared with a paired *t*-test for the variability of IVIM parameters using 10 *b*-values or 4 *b*-values in separate datasets of rBmK AGAP-treated and untreated groups and at different imaging, time points to assess the slightest differences. Within-subject coefficients of variation (wCV) to measure the inconsistency in parameter measurements acquired from the two combinations of *b*-values were presented in percentage and tested using *t*-test. *P <* 0.05 was considered statistically significant.

## Results

### BmK AGAP Inhibited the Growth of Breast Xenograft Tumors

**FIGURE 1:**
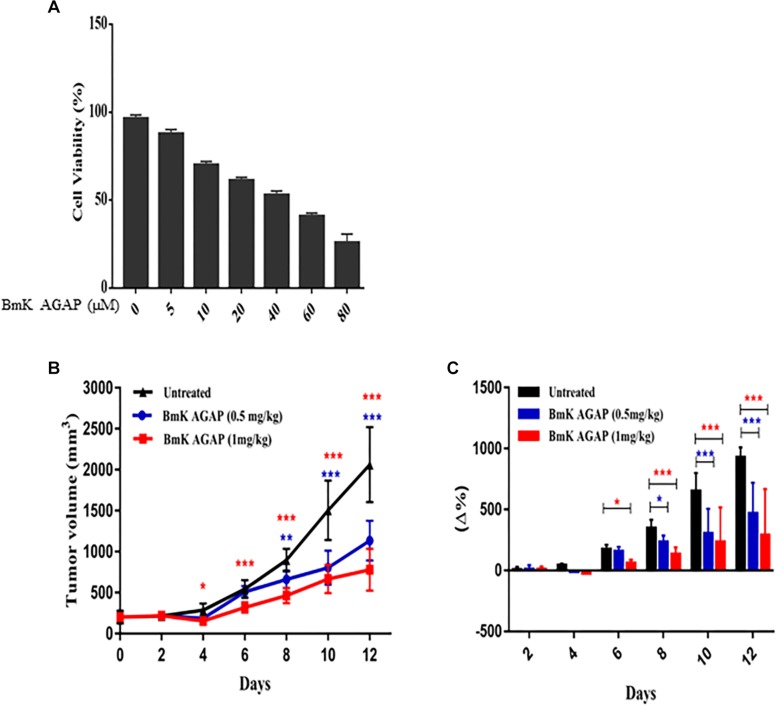
BmK AGAP inhibits the growth of breast xenograft tumors. **(A)** IC_50_ values of rBmK AGAP for MDA-MB-231 cells. Different concentrations of rBmKAGAP were used to treat the cells for 48 h; cell viability was measured by MTT assay. **(B)** Tumor volume of tumors from rBmK AGAP-treated and untreated mice model. Breast xenograft tumor volume was calculated from measuring the length, height, and width of tumors with digital caliper following rBmK AGAP treatment. **(C)** Percentage changes in tumor volumes. The data were statistically significant at ^*^P < 0.05, ^∗∗^*P <* 0.01, and ^∗∗∗^*P <* 0.001 compared with untreated group. The data represent the mean ± SEM of three independent experiments.

To examine the antiproliferation effects of rBmK AGAP on breast cancer with a reduced distribution *b*-values, IVIM-MRI, we first determined the inhibitory concentration value (IC_50_) of rBmK AGAP. The rBmK AGAP inhibited breast cancer cell proliferation in a dose-dependent manner. The viability of MDA-MB-231 cells decreased from 96.3 to 25.6% following rBmK AGAP treatment, ranging between 5 and 80 μM (IC_50_ = 50 μM) for 48 h, compared with untreated cells (Figure 1A).

MDA-MB 231 cells were used to establish a mouse xenograft tumor, and the effects of rBmK AGAP on the tumor growth were assessed. The data showed that average mean tumor volumes varied between 200 and 2,062.08 mm^3^ in the untreated group, whereas in rBmK AGAP (0.5 mg/kg)- and rBmK AGAP (1 mg/kg)-treated groups, the variation fluctuated between 200 and 1,134.15 mm^3^ and between 200 and 779.67 mm^3^, respectively, from day 0 to day 12. The data showed significant difference between rBmK AGAP-treated and untreated groups from day 4 (Figure 1B). We observed a significant change in the percentage of tumor growth rate in the rBmK AGAP (1 mg/kg)-treated mice from day 6 and rBmK AGAP (0.5 mg/kg)-treated mice from day 8 compared with untreated mice. The coefficient of variation gradually increased between 7.6 and 465% in the rBmK AGAP (0.5 mg/kg)-treated mice and between 8.88 and 288.15% in the rBmK AGAP (1 mg/kg)-treated mice compared with 6.5 and 928.02% in the untreated mice from day 4 (Figure 1C). These results suggested that rBmK AGAP inhibits breast cancer proliferation in a dose-dependent manner with a growth rate more than three times slower compared with the untreated tumor.

### MRI Analyses of the Antiproliferative Effects of BmK AGAP on Breast Cancer

**FIGURE 2:**
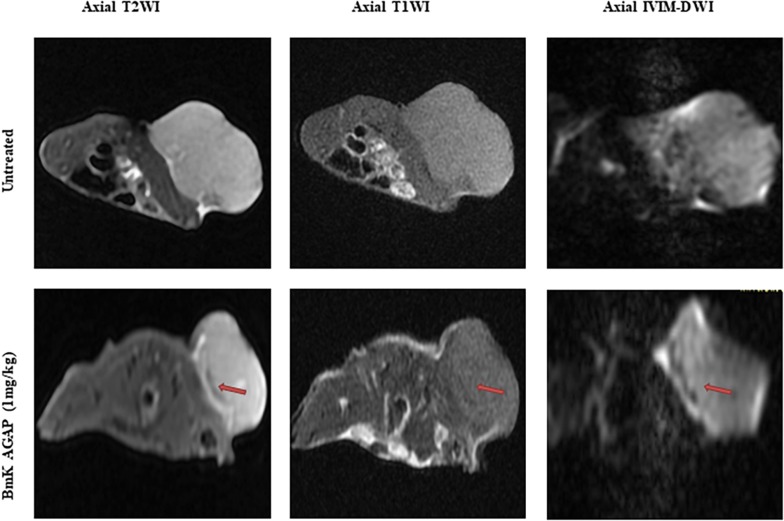
MR images of a tumor of a mouse from the untreated group or the BmK AGAP (1 mg/kg)-treated group, corresponding to day 12 of treatment. Axial T2WIs, axial T1WIs, and axial IVIM images for *b* = 800 s/mm^2^. An area of necrosis is noticed under the sheath (red arrow).

We used unenhanced MRI sequences T1WI and T2WI added to IVIM diffusion sequence to assess the early antiproliferation effects of rBmK AGAP on breast xenograft tumor. The results indicated that tumors obtained from rBmK AGAP-treated mice presented a well-defined and regular margin compared with the untreated tumor. The tumor images acquired on the same day from rBmK AGAP-treated mice showed a heterogeneous distribution of signals discretely increased on T2WI and decreased on T1WI compared with the untreated mice. We also observed an area of necrosis under the tumor sheath obtained from rBmK AGAP-treated mice compared with tumors from the untreated mice (Figure 2).

### Serial Measurements of IVIM Parameters and *ADC* Values in the BmK AGAP-Treated Group and the Untreated Group at Different Time Points

**FIGURE 3:**
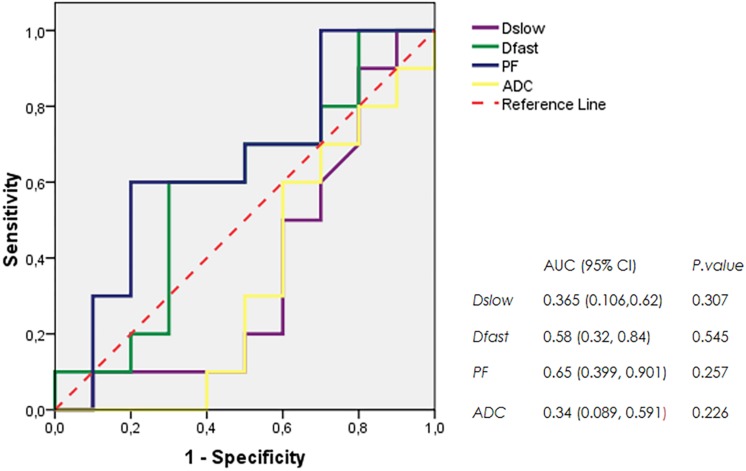
ROC curves generated from *ADC* values and IVIM parameters *Dslow*, *Dfast*, and *PF.*

The mean and SDs of the three IVIM parameters (*Dslow, Dfast*, and *PF*) and *ADC* values at each scanning time point were recorded (Table 1). IVIM parameter values were generally heterogeneously distributed. *Dslow* and *ADC* values increased, and the perfusion parameters (*Dfast* and *PF*) decreased in rBmK AGAP-treated mice. However, the data showed no significant differences in the means between the rBmK AGAP-treated and the untreated mice for each parameter except in *PF* on day 12 *(P* = 0.044*)*. Besides, the ROC curves (Figure 3) displayed an AUC for the IVIM-related parameters *PF* (0.65) larger than that for the other parameters but with a poor medical value (*P* = 0.257).

**TABLE 1:** Serial measurements of IVIM-related parameters and ADC values in the BmK AGAP-treated group and the untreated group at different image acquisition time points (data are presented as means ± SDs and were compared using *t*-test, ^*^P < 0.05).

	**Day 0 (*n* = 6)**	**Day 3 (*n* = 6)**	**Day 6 (*n* = 6)**	**Day 9 (*n* = 6)**	**Day 12 (*n* = 6)**
*Dslow* (10^–3^mm^2^/s)					
Untreated	0.479±0.214	0.83±0.622	0.509±0.105	0.517±0.096	0.617±0.155
BmK AGAP	0.554±0.326	1.098±0.540	0.745±0.418	0.712±0.521	0.745±0.309
*P value*	*0.812*	*0.691*	*0.520*	*0.654*	*0.653*
*Dfast* (10^–3^ mm^2^ /s)					
Untreated	73.5±2.121	31.75±6.151	21.34±18.752	60.5±7.354	73.5±0.99
BmK AGAP	70.55±58.619	49±5.657	24.45±1.485	56.2±2.687	55.5±8.98
*P value*	*0.317*	*0.1*	*0.837*	*0.519*	*0.11*
*PF* (%)					
Untreated	41.55±15.627	25.9±4.808	45.35±9.829	54.65±5.162	61.55±7.001
BmK AGAP	43.85±37.83	14.6±0.283	52.55±5.162	40.585±7.757	37.8±2.121
*P value*	*0.944*	*0.080*	*0.456*	*0.166*	0.044^*^
*ADC* (10^–3^mm^2^/s)					
Untreated	0.515±0.025	0.598±0.058	0.489±0.078	0.428±0.037	0.427±0.117
BmK AGAP	0.563±0.102	0.654±0.302	0.834±0.631	0.687±0.209	0.758±0.484
*P value*	*0.584*	*0.822*	*0.523*	*0.227*	*0.446*

### Assessment of Relative Changes in Measurements of IVIM Parameters Acquired From Combinations of 10 *b*-Values versus 4 *b*-Values

**FIGURE 4:**
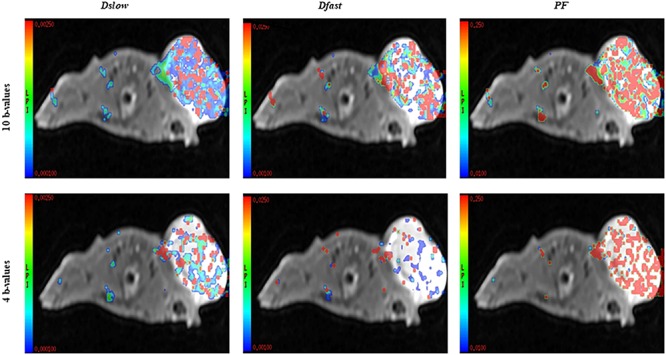
IVIM parametric maps acquired from 10 *b*-values versus 4 *b*-values merged with a T2WI.

**FIGURE 5:**
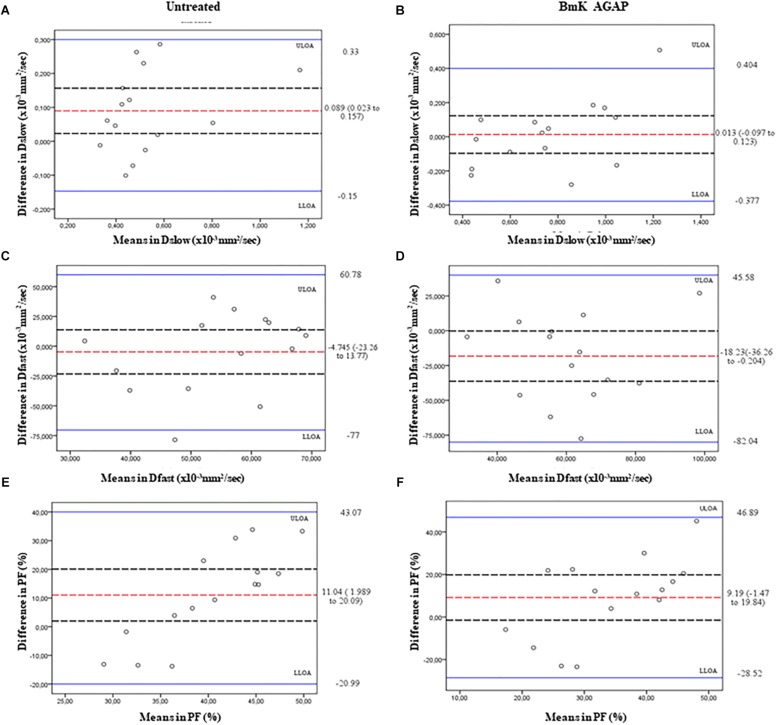
Bland–Altman plots of the parameters measured using the 10 *b*-values versus 4 *b*-values. The average differences (red dashed lines), its 95% confidence intervals (black dashed lines), and upper line of agreement (ULOA), and lower line of agreement (LLOA) (solid blue lines) are displayed. *Dslow* **(A,B)**, *Dfast* **(C,D)**, and *PF* **(E,F)**.

In this study, we compared the parameters acquired from two different combinations of 10 *b*-values and 4 *b*-values. Qualitative analysis of IVIM parametric maps (Figure 4) displayed a loss in signal-to-noise ratio (SNR) that could probably affect the parameter estimations. However, the measurements of *Dslow*, *Dfast*, and *PF* from the two sets of *b*-values at different time points (Table 2) indicated a non-significant variation in parameter measurements, except a case observed in *PF* on the day 3. Table 3 represented the wCV that described the changes within the parameters obtained from either the combinations of 10 *b*-values or 4 *b*-values. Substantial variations were mainly observed in *Dfast* and *PF* at various time points for the rBmK AGAP-treated or the untreated mice. However, no statistically meaningful changes were noticed in *Dslow*, *Dfast*, or *PF* measurements (*P >* 0.05), excluding a single case of significance found in *PF* on day 0. Bland–Altman plots (Figure 5) showed the differences (*y*-axis) and the means (*x*-axis) in parameters measured using the distribution of 10 *b*-values and 4 *b*-values. The systematic bias expressed as average difference between parameters measured using 10 *b*-values and 4 *b*-values for the untreated mice was 0.089 (95% confidence interval: 0.023 to 0.157) in *Dslow*; −4.745 (95% confidence interval: −23.26 to 13.77) in *Dfast*; and 11.04 (95% confidence interval: 1.99 to 20.09) in *PF*. And for the Bmk AGAP-treated mice, the average difference was 0.013 (95% confidence interval: −0.097 to 0.123) in *Dslow*; −18.23 (95% confidence interval: −36.26 to −0.204) in *Dfast*; and 9.19 (95% confidence interval: −1.47 to 19.84) in *PF*. There was no proportional bias among the IVIM parameters acquired (*Dslow*, *Dfast*, and *PF*) for the untreated and BmK-treated mice.

**TABLE 2:** Comparison of IVIM imaging parameters acquired from the combinations of 10 *b*-values and 4 *b*-values in datasets of untreated and BmK AGAP-treated groups and at various image acquisition time points (data are presented as means ± SDs and were compared using paired *t*-tests, ^*^P < 0.05).

	**Day 0 (*n* = 6)**			**Day 3 (*n* = 6)**			**Day 6 (*n* = 6)**			**Day 9 (*n* = 6)**			**Day 12 (*n* = 6)**		
	**10 *b*-values**	**4 *b*-values**	***P*-*value***	**10 *b*-values**	**4 *b*-values**	***P*-*value***	**10 *b*-values**	**4 *b*-values**	***P*-*value***	**10 *b*-values**	**4 b-values**	***P*-*value***	**10 *b*-values**	**4 *b*-values**	***P value***
*Dslow* (10^–3^mm^2^/s)															
Untreated	0.479 ± 0.214	0.37 ± 0.042	*0.533*	0.83 ± 0.622	0.776 ± 0.402	*0.785*	0.509 ± .105	0.535 ± 0.041	*0.664*	0.517 ± 0.096	0.395 ± 0.064	*0.476*	0.617 ± 0.155	0.354 ± 0.029	*0.208*
BmK AGAP	0.554 ± 0.326	0.643 ± 0.132	*0.633*	1.098 ± 0.540	0.985 ± 0.016	*0.821*	0.745 ± 0.418	0.66 ± 0.276	*0.552*	0.712 ± 0.521	0.779 ± 0.497	*0.155*	0.745 ± 0.309	0.722 ± 0.267	*0.585*
*Dfast* (10^–3^ mm^2^/s)															
Untreated	73.5 ± 2.121	51.15 ± 13.506	*0.226*	31.75 ± 6.151	67.35 ± 27.507	*0.255*	21.34 ± 18.752	58.4 ± 39.881	*0.536*	60.5 ± 7.354	43.1 ± 14.001	*0.168*	73.5 ± 0.99	64.55 ± 4.596	*0.265*
BmK AGAP	70.55 ± 58.619	59.25 ± 36.416	*0.603*	49 ± 5.657	74.05 ± 23.688	*0.440*	24.45 ± 1.485	86.35 ± 23.547	*0.157*	56.2 ± 2.687	71.5 ± 40.164	*0.702*	55.5 ± 8.98	55,95 ± 47.588	*0.998*
*PF* (%)																														
Untreated	41.55 ± 15.627	35.1 ± 3.96	*0.578*	25.9 ± 4.808	39.35 ± 5.303	0.017^*^	45.35 ± 9.829	36.0 ± 2.121	*0.336*	54.65 ± 5.162	35.65 ± 3.465	*0.198*	61.55 ± 7.001	27.7 ± 0.424	*0.098*
BmK AGAP	43.85 ± 37.83	33.0 ± 10.607	*0.805*	14.6 ± 0.283	29.05 ± 12.374	*0.340*	52.55 ± 5.162	35.9 ± 0.283	*0.145*	40.585 ± 7.757	24.6 ± 10.889	*0.439*	37.8 ± 2.121	25.6 ± 17.536	*0.313*

**TABLE 3:** wCV in parameters generated from the sets of 10 *b*-values or 4 *b*-values (data were compared using *t*-test, ^*^P < 0.05).

	**Day 0 (*n* = 6)**		**Day 3 (*n* = 6)**		**Day 6 (*n* = 6)**		**Day 9 (*n* = 6)**		**Day 12 (*n* = 6)**	
	**wCV (%)**	***P value***	**wCV (%)**	***P value***	**wCV (%)**	***P value***	**wCV (%)**	***P value***	**wCV (%)**	***P value***
Dslow										
Untreated	17.06	0.449	14.48	0.076	6.59	0.364	18.49	0.474	37.27	0.134
BmK AGAP	20.56	0.423	26.18	0.074	8.06	0.394	9.21	0.368	2.43	0.351
Dfast										
Untreated	26.69	0.265	48.42	0.129	63.44	0.448	21.20	0.242	9.25	0.272
BmK AGAP	14.66	0.198	26.60	0.427	77.81	0.061	31.39	0.204	39.55	0.217
PF										
Untreated	13.51	0.389	29.42	0.053	15.44	0.300	29.63	0.191	53.35	0.066
BmK AGAP	61.91	0.046^*^	42.94	0.264	26.42	0.122	35.69	0.443	34.19	0.477

### Histologic Analyses

**FIGURE 6:**
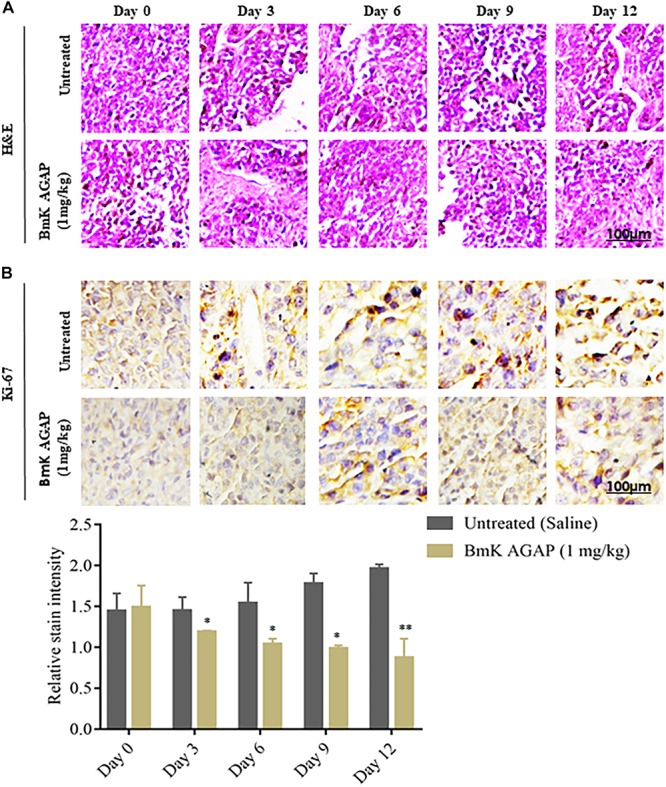
Histologic analyses. **(A)** H&E staining to determine the morphologic changes that formed the basis of the breast xenograft tumor diagnosis. **(B)** Immunohistochemical assessment of proliferation markers in excised tumor tissues. rBmK AGAP (1 mg/kg)-treated xenograft tumor tissues were stained with antibodies against Ki-67 and examined by immunohistochemical staining at different time points (scale bars = 100 μm; magnification, 200x). The data were statistically significant at ^*^P < 0.05 and ^∗∗^*P <* 0.01 as compared with untreated tumors. The data represent the mean ± SD of three independent experiments.

To confirm the observations made from IVIM-MRI, we first performed H&E staining to determine the morphologic changes that formed the basis of the breast xenograft tumor diagnosis (Figure 6A). We then performed immunohistochemical staining to examine the expression of Ki-67 at different time points of the rBmK AGAP (1 mg/kg)-treated mice. The data showed significant decreased expression of Ki-67 in a time-dependent manner following rBmK AGAP treatment compared with the untreated group (Figure 6B). This evidence suggested that BmK AGAP may inhibit proliferation in breast cancer cells.

**FIGURE 7:**
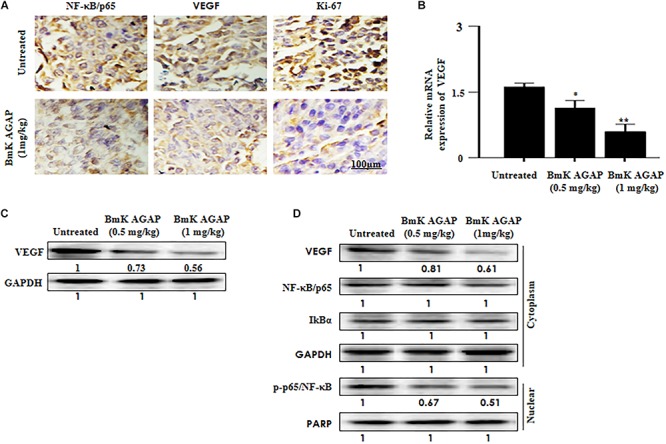
Histologic analyses. VEGF and NF-κB signaling pathway are involved in BmK AGAP inhibition of breast xenograft tumor growth. **(A)** Immunohistochemical assessment of proliferation markers in excised tumor tissues. Xenograft tumor tissues were stained with antibodies against NF-κB/p65, VEGF, and Ki-67 and examined by immunohistochemical staining after day 12 of rBmK AGAP (1 mg/kg) treatment (scale bars = 100 μm; magnification, 200x). **(B)** Relative gene expression of VEGF following rBmK AGAP treatment. Mice were treated with different concentrations of rBmK AGAP for 12 days, and the expression of VEGF and GAPDH (internal control) were analyzed by qPCR. **(C)** VEGF, protein expression following rBmK AGAP treatment of mice bearing breast xenograft tumors. rBmK AGAP-treated tissues were lysed and subjected to 12% SDS-PAGE and analyzed by Western blotting with antibodies against VEGF. **(D)** rBmK AGAP suppresses the expression of VEGF, NF-κB/p65, IκBα, and p-p65/NF-κB in breast xenograft tumors as analyzed by Western blotting. GAPDH or PARP was used as an internal control. The data were statistically significant at ^*^P < 0.05 and ^∗∗^*P <* 0.01 as compared with untreated tumors. The data represent the mean ± SD of three independent experiments.

It is reported that VEGF and NF-κB play essential roles in regulating renal cancer cell proliferation (Djordjević et al., 2008). The expression of VEGF is said to correlate with NF-κB activation in breast cancer (Luo et al., 2016). To investigate the possible mechanism by which BmK AGAP inhibits the growth of breast xenograft tumors, we further performed immunohistochemical staining, qPCR, and Western blot to explore the activity of BmK AGAP on VEGF and activation of NF-κB signaling pathway. VEGF and NF-κB expressions in xenograft tumors were analyzed by tissue immunohistochemical staining. We realized a decreased expression of VEGF and NF-κB, which correlated with decreased Ki-67 expression (Figure 7A). We performed qPCR and Western blot analysis to determine the expression of VEGF in tissue after rBmK AGAP treatment. The qPCR data showed the mRNA of VEGF significantly decreased in a dose-dependent manner following treatment with rBmK AGAP compared with untreated tumors (Figure 7B). Western blot data consistently showed that rBmK AGAP significantly decreased the expression of VEGF and p-p65/ NF-κB in a dose-dependent manner as compared with the untreated group (Figures 7C,D). This evidence suggested that VEGF and the NF-κB signaling pathway are involved in rBmK AGAP inhibition of breast xenograft tumor growth and corroborated with the earlier observation made from the IVIM-MRI analysis of antiproliferation effects of BmK AGAP.

## Discussion

In this study, we aimed to determine the potential of using IVIM-derived parameters to assess early changes induced by BmK AGAP in breast cancer and also to evaluate the variability of the measurements acquired from two different combinations of 10 b-values versus a reduced set of 4 *b*-values. The results showed that the average mean of tumor volume was significantly decreased in the BmK AGAP-treated mice compared with the untreated mice. The relative significant changes in the tumor growth of the BmK AGAP-treated mice were observed from day 4. We also observed that the growth rate of the tumors in BmK AGAP-treated mice was three times decreased at the end of the experiment compared with the untreated mice. The tumor volume decrease was observed on day 4 followed by a gradual but slow increase compared with the untreated group.

Li et al. (2014) reported that BmK AGAP promotes apoptosis and inhibits proliferation of breast cancer cells. Gu et al. (2013) carried out a similar study to demonstrate the antiproliferation effects of BmK AGAP and realized that the analgesic–antitumor peptide BmK AGAP induces apoptosis and inhibits the proliferation of SW480 human colon cancer cells by inactivating the PTEN/PI3K/Akt signaling pathway. Reduced expression of Ki-67 in the BmK AGAP-treated mice in this study revealed noticeable changes in tumors compared with the untreated mice, suggesting a significant therapeutic difference at the cellular level. The slight changes in tumors feature perceived on morphologic MRI sequences should also be considered as modifications in signal, size, margins, and shapes. The presence of necrosis observed in the images from the BmK AGAP-treated mice could describe the effects of the treatment at an early stage, showing the antiproliferative property of the analgesic–antitumor peptide BmK AGAP on breast cancer cells. Our data showed that BmK AGAP inhibited breast tumor growth and suggested that BmK AGAP may be a plausible effective therapeutic approach for cancer.

Vascular endothelial growth factor is one of the major factors that regulate tumor progression and metastatic spread. VEGF is one of the essential signaling proteins involved in vasculogenesis and angiogenesis (Ho and Fong, 2015). NF-κB is a transcriptional factor that plays an important role in the regulation of tumor growth, differentiation, and apoptosis (Sarkar and Li, 2008). Activation of NF-κB results in the induction of a large number of genes involved in the control of a wide variety of biological responses, including VEGF. Abnormal activation of NF-κB is associated with several cancers, including pancreatic cancer, breast cancer, melanoma, and colon cancer (Karim, 2006; Scimeca et al., 2015). The inactivation of NF-κB signaling is linked to decreasing cell growth, apoptosis, and enhanced sensitivity to chemotherapies (Tsai et al., 2015). Kampo et al. (2019) demonstrated that Nav 1.5 functional activities may play a role in BmK AGAP-mediated inactivation of the NF-κB/Wnt/β-catenin signaling pathway to inhibit stemness and epithelial–mesenchymal transition of breast cancer cells *in vitro* and *in vivo* by downregulating PTX3. In this study, decreased expression of VEGF post rBmK AGAP treatment correlated with decreased Ki-67, p65/NF-κB, and NF-κB DNA binding and transcription activity inhibiting breast tumor growth (Figure 7).

An adequate assessment of the efficacy in cancer treatment using medical imaging requires the ability to detect minor changes in biomarkers during the treatment process. Common MRI ways of assessing the response to therapy are DCE-MRI and DWI. Recently, many studies focused on the use of the IVIM bi-exponential model for the same purpose and labeled the non-invasive method to be more accurate than DWI by giving separate diffusion- and perfusion-related parameters (Granata et al., 2015; Lu et al., 2016; Shirota et al., 2016; Lee et al., 2017; Yang et al., 2017). The areas of necrosis might increase in the diffusion-weighted coefficient and perhaps mislead the assessment of the response of a tumor to therapy (Bains et al., 2012). As perfusion is mostly reduced in the areas of necrosis, IVIM diffusion is recommended by several studies as this model gives accurate information about diffusion, taking into consideration the perfusion effect (Le Bihan et al., 1986). In this study, the use of the IVIM DWI MRI sequence to assess the early antiproliferation effect of the analgesic–antitumor peptide BmK AGAP on breast cancer showed significance in the IVIM *PF* parameter on day 12. The relatively late sensitivity of IVIM that involved only one parameter (*PF*) could question the use of IVIM in the assessment of the effect of this drug and might indirectly recommend the use of histologic analyses that seem to give an idea of the effect of the treatment at a very early stage. However, the results call for further studies to assess the BmK AGAP treatment effect using IVIM on a prolonged period of treatment. The parameter *PF* presented the largest AUC on the ROC in comparison to the other parameters though with a poor medical value (AUC = 0.65). This result exposed the sensitivity of the perfusion parameter to the slightest changes in the tumor at the cellular level. Several studies indicated IVIM-related parameters as a potential mean of assessing the efficiency of cancer treatment (Cui et al., 2015; Che et al., 2016; Shirota et al., 2016; Cho et al., 2017; Yang et al., 2017; Kim et al., 2018; Xu et al., 2018).

In the present investigation, we employed the IVIM model for diffusion due to its accurate information about diffusion, taking into consideration the perfusion effect. We observed that the morphological T1WI and T2WI presented some changes related to tumor volume, shape, and signal distribution. However, there was no significance noticed in the IVIM-derived parameters *Dslow*, *Dfast*, and *ADC* values showing the efficiency of the BmK AGAP treatment. Nonetheless, these findings do not discard the efficacy of BmK AGAP treatment on breast cancer since the *PF* showed a relatively late significance on day 12. The ROC revealed that *PF* and *Dfast* have the largest AUC for the prediction of BmK AGAP antiproliferation effects. These findings suggested that the two perfusion-related parameters *PF* and *Dfast* might be potential tools for the assessment of treatment response in cancer. Also, this evidence may emphasize the effective sensitivity of the IVIM perfusion-related parameter at the early stage of BmK AGAP treatment. Similarly, Lee et al. (2017), in an analogous study related to the assessment of a treatment effect on cancer, reported the sensitivity of *PF* and underlined its correlation with microvessel density. Therefore, Cho et al. (2017), in 2017, suggested noticeable variations in *Dfast* as a potential alternative to predict the neoadjuvant treatment effect in breast cancer.

The existing literature describes controversial challenges around the technical parameter setting for the IVIM diffusion model (Iima and Le Bihan, 2016). One of the significant problems is related to the number of *b*-values efficiently used to give accurate results. In our study, we performed a comparative analysis of the IVIM-related parameters generated from a reduced number of *b*-values. Even though the parametric maps displayed a loss in SNR for the set from the 4 *b*-values, the data were generally not different from those of a set from 10 *b*-values. Iima et al. (2018) related similar results out of their investigation by comparing parameters acquired from the 16 *b*-values against those from a set from the 5 *b*-values. These similarities in results could orientate the standardization of the number of *b*-values toward an optimized set that could give accurate information about the diffusivity and the perfusion in living tissues in a non-invasive way and reduce eventually the time of the exam. However, at various time points, the wCVs were generally higher in *Dfast* and *PF* and showed significance in *PF*. Iima et al. (2018) also underlined variability observed in *Dfast*. The authors identified a relative difficulty in the estimation of the pseudodiffusion parameter. In our study, an eventual reason that justifies the inconstancies observed on *Dfast* and *PF* could be the loss in SNR noticed mainly in *Dfast* maps. However, the estimations of the perfusion-related parameters could also be influenced by the quality of low *b*-values included in the reduced distribution.

## Conclusion

In conclusion, these findings suggested that the IVIM parameter *PF* provided significant data that demonstrated the efficacy of the BmK AGAP treatment on breast cancer. Besides, morphologic sequences T1 and T2 displayed some changes confirmed by the substantial changes in immunohistochemistry Ki-67 analysis. This evidence suggested that the analgesic–antitumor peptide BmK AGAP may be a useful therapeutic approach for cancer. *PF*, a perfusion-related parameter of IVIM, could be a valuable tool for further investigations to assess and demonstrate the antiproliferation effect of BmK AGAP on breast cancer within a prolonged period. Despite the inconsistencies observed in *Dfast* and *PF* measurements, a combination of four *b*-values proves the IVIM parameter measurements are beneficial for the assessment of breast cancer tumor responses to BmK AGAP treatment.

## Data Availability

The raw data supporting the conclusions of this manuscript will be made available by the authors, without undue reservation, to any qualified researcher.

## Ethics Statement

The Animal Ethics Committee of the Dalian Medical University, China approved all the experimental animal procedures.

## Author Contributions

ND and SW conceived and designed the study with input from SK and Q-PW. SW, Q-PW, and YL were responsible for the supervision and coordination of the project. ND and SK conducted most of the experiments and were assisted by BA and DZ. ND and SK led the data analysis with inputs from MZ and AM. ND and SK wrote the first draft of the manuscript. YL, BA, DZ, MZ, AM, SW, and Q-PW contributed to the revision and review of the manuscript. All authors read and approved the final manuscript before submission.

## Conflict of Interest Statement

The authors declare that the research was conducted in the absence of any commercial or financial relationships that could be construed as a potential conflict of interest.

## References

[B1] Al-Asmari A. K. Islam M. Al-Zahrani A. M. (2016). In vitro analysis of the anticancer properties of scorpion venom in colorectal and breast cancer cell lines. *Oncol. Lett.* 11 1256–1262. 10.3892/ol.2015.4036 26893728 PMC4734223

[B2] Al-Asmari A. K. Riyasdeen A. Islam M. (2018). Scorpion venom causes apoptosis by increasing reactive oxygen species and cell cycle arrest in MDA-MB-231 and HCT-8 cancer cell lines. *J. Evid. Based Integr. Med.* 23:2156587217751796. 29405760 10.1177/2156587217751796 PMC5881405

[B3] Al-Mahmood S. Sapiezynski J. Garbuzenko O. B. Minko T. (2018). Metastatic and triple-negative breast cancer: challenges and treatment options. *Drug Deliv. Transl. Res.* 5 1483–1507. 10.1007/s13346-018-0551-3 29978332 PMC6133085

[B4] Bains L. J. Zweifel M. Thoeny H. C. (2012). Therapy response with diffusion MRI: an update. *Cancer Imaging* 12 395–402. 10.1102/1470-7330.2012.9047 23022595 PMC3460562

[B5] Che S. Zhao X. Ou Y. Li J. Wang M. Wu B. (2016). Role of the intravoxel incoherent motion diffusion weighted imaging in the pre-treatment prediction and early response monitoring to neoadjuvant chemotherapy in locally advanced breast cancer. *Medicine* 95:e2420. 10.1097/MD.0000000000002420 26825883 PMC5291553

[B6] Cho G. Y. Gennaro L. Sutton E. J. Zabor E. C. Zhang Z. Giri D. (2017). Intravoxel incoherent motion (IVIM) histogram biomarkers for prediction of neoadjuvant treatment response in breast cancer patients. *Eur. J. Radiol. Open* 4 101–107. 10.1016/j.ejro.2017.07.002 28856177 PMC5565789

[B7] Cho G. Y. Moy L. Zhang J. L. Baete S. Lattanzi R. Moccaldi M. (2015). Comparison of fitting methods and b-value sampling strategies for intravoxel incoherent motion in breast cancer. *Magn. Reson. Med.* 74 1077–1085. 10.1002/mrm.25484 25302780 PMC4439397

[B8] Cui Y. Zhang C. Li X. Liu H. Yin B. Xu T. (2015). Intravoxel incoherent motion diffusion-weighted magnetic resonance imaging for monitoring the early response to ZD6474 from nasopharyngeal carcinoma in nude mouse. *Sci. Rep.* 5:16389. 10.1038/srep16389 26574153 PMC4648100

[B9] Cui Y. Zhang X. P. Sun Y. S. Tang L. Shen L. (2008). Apparent diffusion coefficient: potential imaging biomarker for prediction and early detection of response to chemotherapy in hepatic metastases. *Radiology* 248 894–900. 10.1148/radiol.2483071407 18710982

[B10] Djordjević G. Matusan-Ilijas K. Sinozić E. Damante G. Fabbro D. Grahovac B. (2008). Relationship between vascular endothelial growth factor and nuclear factor-kappaB in renal cell tumors. *Croat Med. J.* 49 608–617. 10.3325/cmj.2008.5.608 18925694 PMC2582353

[B11] Fan G. Zang P. Jing F. Wu Z. Guo Q. (2005). Usefulness of diffusion/perfusion-weighted MRI in rat gliomas: correlation with histopathology. *Acad. Radiol.* 12 640–651. 10.1016/j.acra.2005.01.024 15866139

[B12] Faustino-Rocha A. Oliveira P. A. Pinho-Oliveira J. Teixeira-Guedes C. Soares-Maia R. da Costa R. G. (2013). Estimation of rat mammary tumor volume using caliper and ultrasonography measurements. *Lab. Anim.* 42 217–224. 10.1038/laban.254 23689461

[B13] Granata V. Fusco R. Catalano O. Filice S. Amato D. M. Nasti G. (2015). Early assessment of colorectal cancer patients with liver metastases treated with antiangiogenic drugs: the role of intravoxel incoherent motion in diffusion-weighted imaging. *PLoS One* 10:e0142876. 10.1371/journal.pone.0142876 26566221 PMC4643930

[B14] Gu Y. Liu S.-L. Ju W.-Z. Li C.-Y. Cao P. (2013). Analgesic–antitumor peptide induces apoptosis and inhibits the proliferation of SW480 human colon cancer cells. *Oncol. Lett.* 5 483–488. 10.3892/ol.2012.1049 23420047 PMC3573048

[B15] Guo G. Cui Y. Chen H. Zhang L. Zhao M. Chen B. (2016). Analgesic–antitumor peptide inhibits the migration and invasion of HepG2 cells by an upregulated VGSC β1 subunit. *Tumor Biol.* 37 3033–3041. 10.1007/s13277-015-4067-x 26419595

[B16] Guo Y. Cai Y. Q. Cai Z. L. Gao Y. G. An N. Y. Ma L. (2002). Differentiation of clinically benign and malignant breast lesions using diffusion-weighted imaging. *J. Magn. Reson. Imaging* 16 172–178. 10.1002/jmri.10140 12203765

[B17] Ho V. C. Fong G. H. (2015). Vasculogenesis and angiogenesis in VEGF receptor-1 deficient mice. *Methods Mol. Biol.* 1332 161–176. 10.1007/978-1-4939-2917-7_12 26285753 PMC4544766

[B18] Humphries P. D. Sebire N. J. Siegel M. J. Olsen E. (2007). Tumors in pediatric patients at diffusion-weighted MR imaging: apparent diffusion coefficient and tumor cellularity. *Radiology* 245 848–854. 10.1148/radiol.2452061535 17951348

[B19] Iima M. Kataoka M. Kanao S. Kawai M. Onishi N. Koyasu S. (2018). Variability of non-Gaussian diffusion MRI and IVIM measurements in the breast. *PLoS One* 13:e0193444. 10.1371/journal.pone.0193444 29494639 PMC5832256

[B20] Iima M. Le Bihan D. (2016). Clinical intravoxel incoherent motion and diffusion MR imaging: past, present, and future. *Radiology* 278 13–32. 10.1148/radiol.2015150244 26690990

[B21] Iima M. Reynaud O. Tsurugizawa T. Ciobanu L. Li J. R. Geffroy F. (2014). Characterization of glioma microcirculation and tissue features using intravoxel incoherent motion magnetic resonance imaging in a rat brain model. *Invest. Radiol.* 49 485–490. 10.1097/RLI.0000000000000040 24619211

[B22] Kampo S. Ahmmed B. Zhou T. Owusu L. Anabah T. W. Doudou N. R. (2019). Scorpion venom analgesic peptide, BmK AGAP inhibits stemness and epithelial-mesenchymal transition by down-regulating PTX3 in breast cancer. *Front. Oncol.* 9:21. 10.3389/fonc.2019.00021 30740360 PMC6355678

[B23] Karim M. (2006). Nuclear factor-kB in cancer development and progression. *Nature* 441 431–436. 10.1038/nature04870 16724054

[B24] Karlsson E. Appelgren J. Solterbeck A. Bergenheim M. Alvariza V. Bergh J. (2014). Breast cancer during follow-up and progression—a population based cohort on new cancers and changed biology. *Eur. J. Cancer* 50 2916–2924. 10.1016/j.ejca.2014.08.014 25241230

[B25] Kennecke H. Yerushalmi R. Woods R. Cheang M. C. Voduc D. Speers C. H. (2010). Metastatic behavior of breast cancer subtypes. *J. Clin. Oncol.* 28 3271–3277. 10.1200/JCO.2009.25.9820 20498394

[B26] Kim Y. Kim S. H. Lee H. W. Song B. J. Kang B. J. Lee A. (2018). Intravoxel incoherent motion diffusion-weighted MRI for predicting response to neoadjuvant chemotherapy in breast cancer. *Magn. Reson. Imaging* 48 27–33. 10.1016/j.mri.2017.12.018 29278762

[B27] Koh D. M. Scurr E. Collins D. Kanber B. Norman A. Leach M. O. (2007). Predicting response of colorectal hepatic metastasis: the value of pretreatment apparent diffusion coefficients. *AJR Am. J. Roentgenol.* 188 1001–1008. 10.2214/AJR.06.0601 17377036

[B28] Le Bihan D. Breton E. Lallemand D. Grenier P. Cabanis E. Laval-Jeantet M. (1986). MR imaging of intravoxel incoherent motions: application to diffusion and perfusion in neurologic disorders. *Radiology* 161 401–407. 10.1148/radiology.161.2.3763909 3763909

[B29] Lee Y. Lee S. S. Cheong H. Lee C. K. Kim N. Son W. C. (2017). Intravoxel incoherent motion MRI for monitoring the therapeutic response of hepatocellular carcinoma to sorafenib treatment in mouse xenograft tumor models. *Acta Radiol.* 58 1045–1053. 10.1177/0284185116683576 28273738

[B30] Li W. Li Y. Zhao Y. Yuan J. Mao W. (2014). Inhibition effects of scorpion venom extracts (Buthus matensii Karsch) on the growth of human breast cancer MCF-7 cells. *Afr. J. Tradit. Complement. Altern. Med.* 11 105–110. 25395713 10.4314/ajtcam.v11i5.17 PMC4202526

[B31] Liedtke C. Mazouni C. Hess K. R. André F. Tordai A. Mejia J. A. (2008). Response to neoadjuvant therapy and long-term survival in patients with triple-negative breast cancer. *J. Clin. Oncol.* 26 1275–1281. 10.1200/JCO.2007.14.4147 18250347

[B32] Liu X. Liu X. Sunchen S. Liu M. Shen C. Wu J. (2017). A novel tumor-activated ALA fusion protein for specific inhibition on the growth and invasion of breast cancer cells MDA-MB-231. *Drug Deliv.* 24 1811–1817. 10.1080/10717544.2017.1406560 29172777 PMC8241173

[B33] Lu L. Li Y. Li W. (2016). The role of intravoxel incoherent motion MRI in predicting early treatment response to chemoradiation for metastatic lymph nodes in nasopharyngeal carcinoma. *Adv. Ther.* 33 1158–1168. 10.1007/s12325-016-0352-3 27294489

[B34] Luo M. Hou L. Li J. Shao S. Huang S. Meng D. (2016). VEGF/NRP-1axis promotes progression of breast cancer via enhancement of epithelial–mesenchymal transition and activation of NF-κB and β-catenin. *Cancer Lett.* 373 1–11. 10.1016/j.canlet.2016.01.010 26805761

[B35] Ma R. Cui Y. Zhou Y. Bao Y.-M. Yang W.-Y. Liu Y.-F. (2010). Location of the analgesic domain of scorpion toxin BmK AGAP by mutagenesis of disulfide bridges. *Biochem. Biophys. Res. Commun.* 394 330–334. 10.1016/j.bbrc.2010.02.179 20206129

[B36] Moore-Smith L. Forero-Torres A. Stringer-Reasor E. (2018). Future developments in neoadjuvant therapy for triple-negative breast cancer. *Surg. Clin. North Am.* 98 773–785. 10.1016/j.suc.2018.04.004 30005773

[B37] Patterson D. M. Padhani A. R. Collins D. J. (2008). Technology insight: water diffusion MRI—a potential new biomarker of response to cancer therapy. *Nat. Clin. Pract. Oncol.* 5 220–233. 10.1038/ncponc1073 18301415

[B38] Robba T. Chianca V. Albano D. Clementi V. Piana R. Linari A. (2017). Diffusion-weighted imaging for the cellularity assessment and matrix characterization of soft tissue tumor. *Radiol. Med.* 122 871–879. 10.1007/s11547-017-0787-x 28689283

[B39] Rodríguez Bautista R. Ortega Gómez A. Hidalgo Miranda A. Zentella Dehesa A. Villarreal-Garza C. Ávila-Moreno F. (2018). Long non-coding RNAs: implications in targeted diagnoses, prognosis, and improved therapeutic strategies in human non- and triple-negative breast cancer. *Clin. Epigenet.* 10:88. 10.1186/s13148-018-0514-z 29983835 PMC6020372

[B40] Sarfo-Poku C. Eshun O. Lee K. H. (2016). Medical application of scorpion venom to breast cancer: a mini-review. *Toxicon* 122 109–112. 10.1016/j.toxicon.2016.09.005 27644898

[B41] Sarkar F. H. Li Y. (2008). NF-kappaB: a potential target for cancer chemoprevention and therapy. *Front. Biosci.* 13:2950–2959. 10.2741/2900 17981768

[B42] Scimeca M. Antonacci C. Colombo D. Bofiglio R. Buonomo O. C. Bonanno E. (2015). Emerging prognostic markers related to mesenchymal characteristics of poorly differentiated breast cancer. *Tumor Biol.* 4 5427–5435. 10.1007/s13277-015-4361-7 26563370

[B43] Shirota N. Saito K. Sugimoto K. Takara K. Moriyasu F. Tokuuye K. (2016). Intravoxel incoherent motion MRI as a biomarker of sorafenib treatment for advanced hepatocellular carcinoma: a pilot study. *Cancer Imaging* 16:1. 10.1186/s40644-016-0059-3 26822946 PMC4731920

[B44] Siegel R. L. Miller K. D. Jemal A. (2017). Cancer statistics. *CA Cancer J. Clin.* 67 7–30. 10.3322/caac.21387 28055103

[B45] Sugahara T. Korogi Y. Kochi M. Ikushima I. Shigematu Y. Hirai T. (1999). Usefulness of diffusion-weighted MRI with echo-planar technique in the evaluation of cellularity in gliomas. *J. Magn. Reson. Imaging* 9 53–60. 10.1002/(sici)1522-2586(199901)9 10030650

[B46] Tong C. W. S. Wu M. Cho W. C. S. To K. K. W. (2018). Recent advances in the treatment of breast cancer. *Front. Oncol.* 8:227. 10.3389/fonc.2018.00227 29963498 PMC6010518

[B47] Tsai J. R. Liu P. L. Chen Y. H. Chou S. H. Cheng Y. J. Hwang J. J. (2015). Curcumin inhibits non-small cell lung cancer cells metastasis through the adiponectin/NF-kB/MMPs signaling pathway. *PLoS One* 10:e014442. 10.1371/journal.pone.0144462 26656720 PMC4675518

[B48] Turkbey B. Shah V. P. Pang Y. Bernardo M. Xu S. Kruecker J. (2011). Is apparent diffusion coefficient associated with clinical risk scores for prostate cancers that are visible on 3-T MR images? *Radiology* 258 488–495. 10.1148/radiol.10100667 21177390 PMC3029887

[B49] Xu Q. Xu Y. Sun H. Chan Q. Shi K. Song A. (2018). Quantitative intravoxel incoherent motion parameters derived from the whole-tumor volume for assessing complete pathological response to neoadjuvant chemotherapy in locally advanced rectal cancer. *J. Magn. Reson. Imaging* 48 248–258. 10.1002/jmri.25931 29281151

[B50] Yamashita Y. Kumabe T. Higano S. Watanabe M. Tominaga T. (2009). Minimum apparent diffusion coefficient is significantly correlated with cellularity in medulloblastomas. *Neurol. Res.* 31 940–946. 10.1179/174313209X382520 19138469

[B51] Yang S. H. Lin J. Lu F. Han Z. H. Fu C. X. Lv P. (2017). Evaluation of antiangiogenic and antiproliferative effects of sorafenib by sequential histology and intravoxel incoherent motion diffusion-weighted imaging in an orthotopic hepatocellular carcinoma xenograft model. *J. Magn. Reson. Imaging* 45 270–280. 10.1002/jmri.25344 27299302

[B52] Zhao Y. Cai X. Ye T. Huo J. Liu C. Zhang S. (2011). Analgesic–antitumor peptide inhibits proliferation and migration of SHG-44 human malignant glioma cells. *J. Cell Biochem.* 112 2424–2434. 10.1002/jcb.23166 21538480

[B53] Zhuang Z. Zhang Q. Zhang D. Cheng F. Suo S. Geng X. (2018). Utility of apparent diffusion coefficient as an imaging biomarker for assessing the proliferative potential of invasive ductal breast cancer. *Clin. Radiol.* 73 473–478. 10.1016/j.crad.2017.11.019 29273228

